# Supplementary material for: Impact of OsBadh2 Mutations on Salt Stress Response in Rice
Source: Plants (Basel). 2022 Oct 24;11(21):2829. doi: 10.3390/plants11212829 (PMC9656462; doi:10.3390/plants11212829)
Supplement: Supplementary file 1 [file plants-11-02829-s001.zip › plants-1956884-supplementary.pdf]

plants

MDPI

Supplementary Table S1: List of primers used in this study.

Name of Primer	Gene ID/Name	Sequence	Remarks
BADH2.7PCRF	*LOC_Os08g32870*	CTTGTTTGGAGCTTGCTGATG	*OsBadh2* gene sequence
BADH2.7PCRR		AGTGAAACAGGCTGTCAAGAA	
BAD2SgF		GGCAGACTAGAGACGCTTGATTGT	*OsBadh2* gene guideRNA
BAD2SgR		AAACACAATCAAGCGTCTCTAGTC	
Hyg-F		AGAAGAAGATGTTGGCGACCT	Hygromycin marker primer
Hyg-R		GTCCTGCGGGTAAATAGCT	
Vec-seq F	PHUN4c12	GCCCATTACGCAATTGGACG	To confirm oligos insertion in pHUN4c12
Vec-seq R		CAAGGACGACGGCAACTACA	
OsBadh1F	*LOC_Os04g39020*	CCACGGAGGCAACCATC	RTqPCR primers
OsBadh1R		CCTTGAGGTACTTGGCCC	
OsBadh2F	*LOC_Os08g32870*	CTTGTTTGGAGCTTGCTGATG	
OsBadh2R		CATAACTCCCAGTAAATGCAACC	
OsP5CS1F	*LOC_Os05g38150*	CAAGATGGAAGATTGGCTTTGG	
OsP5CS1R		GTACCTAAGTCGCTGTCGC	
OsP5CS2F	*LOC_Os01g62900*	TGCTGTTGGTGTTGGGAG	
OsP5CS2R		AACGTGTCATAGATCGCCATC	
OsGAD1F	*LOC_Os08g36320*	CTGATGCTGGACGGGAAC	
OsGAD1R		TCCATGTCGACGTAGTTCTTG	
OsGAD2F	*LOC_Os04g37500*	ACAAGAACTACGCCGACATG	
OsGAD2R		CATTGAACAGCCTCGCTATG	
OsGAD3F	*LOC_Os03g13300*	CGTCTCCGAGAGTGACATG	
OsGAD3R		TTGCCGTCCAGCATCAG	
OsGAD4F	*LOC_Os03g51080*	CACTCCACGTTCGCCTC	
OsGAD4R		TTGCCGTCCAGCATCAG	
OsNAC3F	*LOC_Os07g12340*	GAAGAACGAGTGGGAGAAGATG	
OsNAC3R		GCGAGCATGGAGAGGTC	
OsMYB30F	*LOC_Os02g41510*	GTGGATCAACTACCTCCGC	
OsMYB30R		TTCTTGATCTCGTTGTCCGTC	
OsDREB1CF	*LOC_Os06g03670*	CAAGTTCAGGGAGACGAGG	
OsDREB1CR		AGATGCGGGACTTCTTGTTG	
OsERF068F	*LOC_Os01g21120*	TCATCTACGACTACATCCCGG	
OsERF068R		GTTCTTCCGCTCCCTCTTC	
OsHAK5F	*LOC_Os01g70490*	CCAAAGCCATACAGCCAAG	
OsHAK5R		TCCTTGATCCCGTTGGTAAAG	
ActinF	*LOC_Os08g28190*	CGTGAATACCAGCTAGGACTTG	
ActinR		TCAGGAGCTTGAAACCGTTC	
